# ‘*Making the Invisible Visible*’: an audience response to an art installation representing the complexity of congenital heart disease and heart transplantation

**DOI:** 10.1136/medhum-2018-011466

**Published:** 2018-10-18

**Authors:** Giovanni Biglino, Sofie Layton, Matthew Lee, Froso Sophocleous, Susannah Hall, Jo Wray

**Affiliations:** 1 Translational Health Sciences, Bristol Medical School, Bristol Heart Institute, University of Bristol, Bristol, UK; 2 GOSH Arts, Great Ormond Street Hospital for Children NHS Foundation Trust, London, UK; 3 Bristol Medical School, University of Bristol, Bristol, UK; 4 Cardiorespiratory Division, Great Ormond Street Hospital for Children NHS Foundation Trust, London, UK

**Keywords:** art and medicine, cardiology, paediatrics, patient narratives

## Abstract

The arts can aid the exploration of individual and collective illness narratives, with empowering effects on both patients and caregivers. The artist, partly acting as conduit, can translate and re-present illness experiences into artwork. But how are these translated experiences received by the viewer—and specifically, how does an audience respond to an art installation themed around paediatric heart transplantation and congenital heart disease? The installation, created by British artist Sofie Layton and titled *Making the Invisible Visible*, was presented at an arts-and-health event. The piece comprised three-dimensional printed medical models of hearts with different congenital defects displayed under bell jars on a stainless steel table reminiscent of the surgical theatre, surrounded by hospital screens. The installation included a soundscape, where the voice of a mother recounting the journey of her son going through heart transplantation was interwoven with the voice of the artist reading medical terminology. A two-part survey was administered to capture viewers’ expectations and their response to the piece. Participants (n=125) expected to acquire new knowledge around heart disease, get a glimpse of patients’ experiences and be surprised by the work, while after viewing the piece they mostly felt empathy, surprise, emotion and, for some, a degree of anxiety. Viewers found the installation more effective in communicating the experience of heart transplantation than in depicting the complexity of cardiovascular anatomy (p<0.001, z=7.56). Finally, analysis of open-ended feedback highlighted the intimacy of the installation and the privilege viewers felt in sharing a story, particularly in relation to the soundscape, where the connection to the narrative in the piece was reportedly strengthened by the use of sound. In conclusion, an immersive installation including accurate medical details and real stories narrated by patients can lead to an empathic response and an appreciation of the value of illness narratives.

## Introduction

The heart has taken several disguises across religion, art and literature,^1^ from the haunting heartbeat symbolising remorse and guilt in Poe to ‘a lonely hunter that hunts on a lonely hill’ according to Scottish Romantic poet Fiona Macleod and reprised famously by Carson McCullers.

Artistic representations of the heart as a symbol range from the visual, for example, religious ex-voto imagery but also contemporary depictions by the likes of Keith Haring or Banksy, to large-scale installations, for example, Indian contemporary artist Bharti Kher created a huge fibreglass heart in her piece *An Absence of Assignable Cause* (2007), encapsulating the whole anatomy of a blue sperm whale in the heart (arguably the core of an organism) and decorating the heart and its protruding arteries and veins with *bindis*, the forehead decoration worn by Indian women.^2^ However, beyond the anatomy of the heart and its rich symbolism, the *experience of heart disease* is explored to a somewhat more limited degree artistically.

Indeed, the artistic medium can be used bothautobiographically, to explore one’s own experience of heart disease, and to represent others’ illness experiences. New York artist Victoria Behm, for example, created a pictorial journal to come to terms with her diagnosis of congestive heart failure and living with a wearable defibrillator.^3^ Artist Florence Hawkins-Criss found inspiration for a series of paintings (including ECG electrodes) from the experience of wearing a heart monitor for 3 weeks.^4^ British artist Alexa Wright, on the other hand, created an interactive sound installation (incorporating patients’ narratives) to explore how heart transplant can impact on the recipient’s sense of self and individuality.^5^ Finally, British artist Sofie Layton’s practice has recently explored how a participatory arts process can lead to aiding young people with congenital heart disease to explore their individual uniqueness^6^ as well as creating original artworks encapsulating their families’ and their own stories.^7^

But how are such representations received and perceived by a viewer—and, specifically, how does an audience respond to an art installation themed around paediatric heart transplantation and congenital heart disease?

### Ways of seeing and narratives of illness

While discussion of heart disease has been reported to involve a limited use of metaphors compared with other conditions such as cancer or HIV/AIDS, as observed by essayist Susan Sontag who discussed how clusters of metaphors influence discourse around and public attitudes towards diseases,^8 9^ generally illnesses are not metaphor free, despite technological advances.^10^ Biomedical narrative is limited in its power to convey full meanings of illness experiences and treatments, hence the need to express nuances of illness experiences through metaphors.

The arts can aid the delicate exploration of individual illness experiences.^11^ It is increasingly recognised that an artistic approach (with an interdisciplinary component) can convey illness experiences which otherwise would be inaccessible, such as, for example, the experience of haematopoietic stem cell transplant as treatment for acute leukaemia.^12^ It can have empowering effects on both patients and caregivers.

Arts practices in healthcare contexts are distinct from arts therapy, and the context itself has been defined as ‘a nebulous complex’^13^ and characterised by a tension between clinical evidence-based practice versus contemporary arts practice. Such complexity is reflected in the multiplicity of meanings that the arts can have—for patients, for clinicians and also for artists themselves. Empathy, conviviality, clinical outcomes and aesthetic visions all coexist in this space. In a medical context, the artist observes and witnesses forms, language, conversations and interactions, and can channel them; but the artist in this context is likely a witness, and has their own emotional response to the subject matter.^14^ The emerging artistic response and arts-informed research have been described as having a positive impact on the audience, resulting in increased engagement in the conversation around illness experiences and also increased awareness around illness narratives.^15–17^

Within this broad framework, our aim was to use qualitative and quantitative methods to address our research question and assess an audience’s response to an art installation themed around paediatric heart transplantation and congenital heart disease.

## Materials and methods

### Installation and setting

The installation, titled *Making the Invisible Visible*, was created by artist Sofie Layton in 2015–2016 during a 1 year residency at Great Ormond Street Hospital for Children in London. Stemming from participatory work undertaken by the artist and the team,^6 18^ the piece explored the experience of living with congenital heart disease and of undergoing heart transplantation, from the perspective of the patients and their families. The artist created a landscape of heart models, which were 1:1 replicas of the hearts of patients with different types of congenital heart disease (including tetralogy of Fallot, total anomalous pulmonary venous drainage, congenital aortic stenosis, transposition of the great arteries and hypoplastic left heart syndrome). The models were derived from cardiovascular MRI data sets, reconstructed in three dimensions (3D) following a validated methodology,^19^ and then manufactured by means of 3D printing technology (Rapidform, Royal College of Art, London, UK). The heart models were previously used in some of our previous medical studies,^20 21^ following appropriate ethical protocols. All patients and/or their parents provided written consent for their use in our work, including in exhibitions. Although overtly medical as objects, the 3D heart models were placed under glass bell jars, conferring to them the aura of museum artefacts. The bell jars were placed on a stainless steel table, reminiscent of the surgical theatre, which in turn was surrounded by fine white voile curtained hospital screens. The voile panels of the hospital screens were screen printed—white on white—with ECG tracings and other cardiac-themed references. Finally, the installation was completed by a soundscape, created by Layton in collaboration with sound composer Jules Maxwell. The soundscape consisted of a mother recounting her son’s heart transplantation and the voice of the child himself, interwoven with the voice of the artist reading medical jargon and technical anatomical/medical terms, that is, the voice of the patient and the carer mixing with the technical language pertaining to cardiovascular disease and its treatment. The mother provided written informed consent for the recording and use of the soundscape and the child assented to his voice being used as part of this work. The soundscape opened with a brief excerpt of church music, evocative of a prior installation of this artwork in a church setting and referencing also the religious symbolism of the heart.

**Figure 1:**
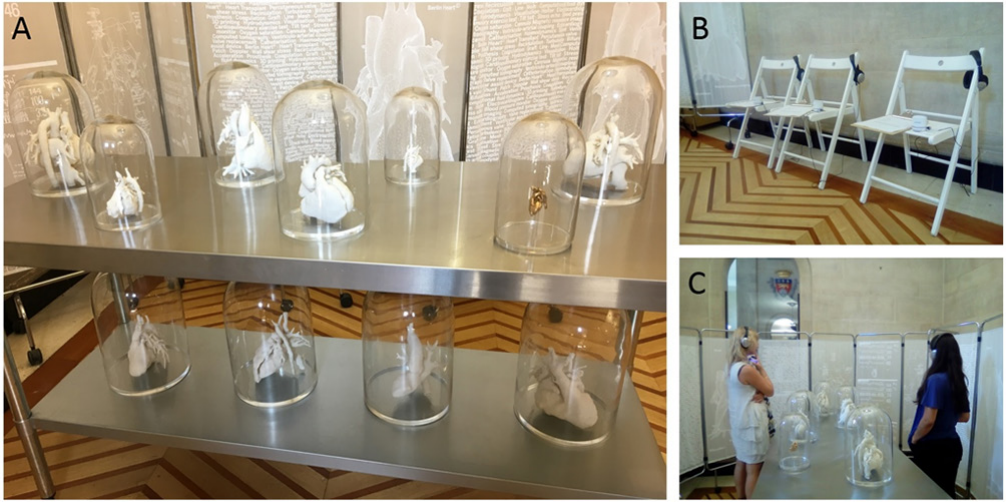
(A) Installation of *Making the Invisible Visible* in Bristol City Hall, showing the medical models under bell jars displayed on a steel table reminiscent of the surgical theatre and surrounded by hospital screens. (B) Visitors were provided with headphones to listen to the soundscape that accompanied the piece. (C) Visitors could sit while listening to the soundscape or walk around the table and view the heart models at the same time.

**Figure 2:**
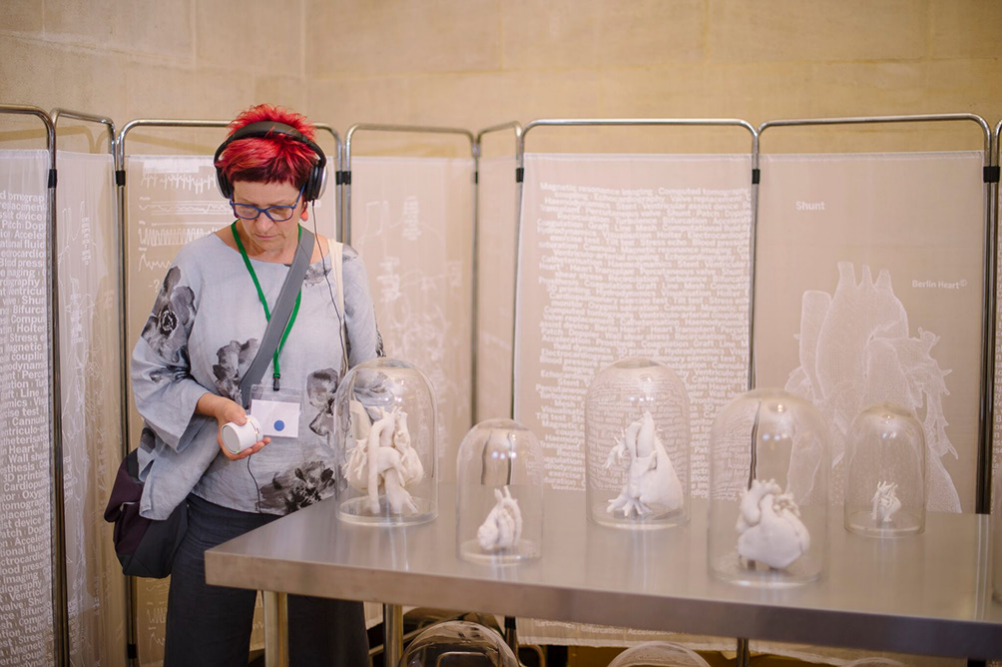
Visitors could listen to the soundscape on headphones for a more immersive experience. (Image by Jim Wileman)

**Figure 3:**
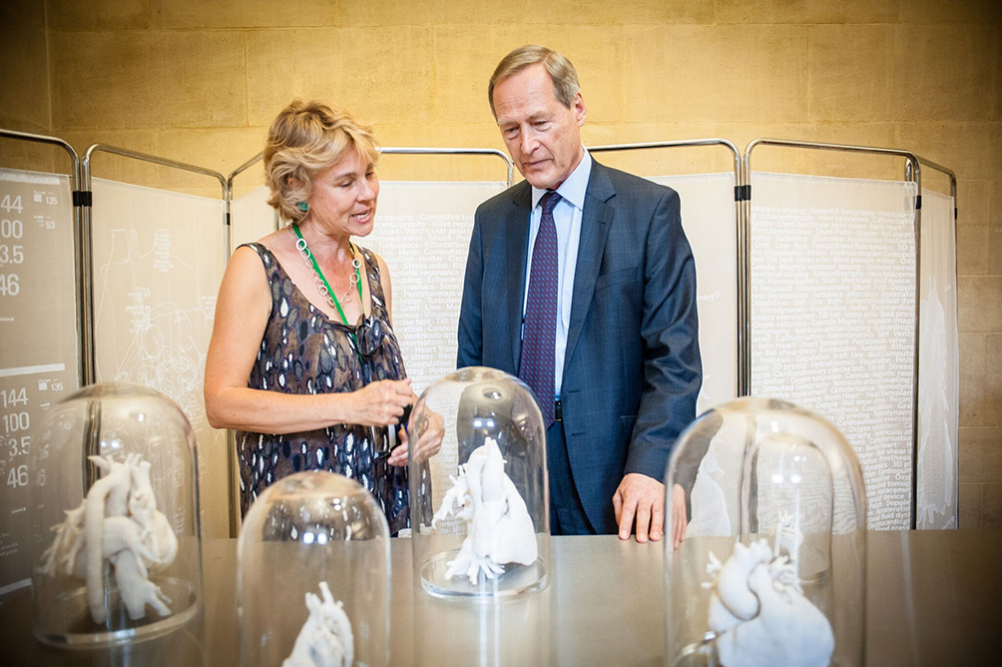
Visitors also had the opportunity to directly discuss the piece with the artist. (Image by Clint Randall)

This was the third reconfiguration of *Making the Invisible Visible*, which had been previously shown at Great Ormond Street Hospital/Institute of Child Health in London (10 February to 10 April 2016)^22^ and then, as part of the *Feel It Festival*, at St Paul’s Church in Bristol (17–20 November 2016).^23^ The installation was not substantially changed from previous configurations, but adapted to fit the current setting for the piece, that is, the main foyer of Bristol City Hall. The installation was exhibited for the duration of the *Culture, Health and Wellbeing International Conference* (Bristol, 19–21 June 2017). A short leaflet containing basic information about the installation was designed specifically for the event and given to delegates on their arrival. The installation was also publicised in the conference programme and on the website, and delegates were given the opportunity to book a slot in advance to view the installation at specific times. The setting (figure 1) included mp3 players and headphones for viewers to listen to the soundscape while viewing the installation. The headphones (figure 2) ensured a more immersive and concentrated experience than more dispersive ambient sound. Chairs were available for conference delegates, as well as other viewers entering City Hall, to listen to the soundscape and observe the piece, or they also had the freedom to walk around the table and navigate the landscape at their own pace. The duration of the soundscape was approximately 7 min. After listening to the soundscape and completing the survey, visitors also had the opportunity to discuss directly with the artist (figure 3).

### Evaluation of the audience’s response

All conference delegates approaching and viewing the installation were consecutively invited to complete a two-part questionnaire designed to evaluate their response to the installation. The brief survey was developed by the team under the guidance of a psychologist (JW) and included a mix of rating scales and free-text questions. It was administered before and after a viewer had experienced the piece, including the soundscape. Two researchers were always present, invigilating the artwork and collecting questionnaires from the viewers, as well as helping them to set up the headphones and answering any questions they may have on the genesis of the piece or on the 3D heart models.

In the first part of the questionnaire, in order to evaluate the expectations that a viewer may have when approaching an installation themed around a medical condition, participants were asked to provide three words, phrases or sentences summarising their expectations. Further audience information (eg, gender or professional status) was collected but could not in the end be used due to the small sample size.

The second part of the questionnaire was completed after the viewer had experienced the installation and included quantitative and qualitative questions to assess the impact the installation had on them. First, viewers were asked for three words, phrases or sentences to summarise their feelings having experienced the installation, mirroring the question on expectations in part 1 of the questionnaire. Second, they were asked how effective the installation was in (A) conveying the experience of heart transplantation and (B) describing heart anatomy and heart disease, on a Likert scale from 0 (not effective at all) to 10 (extremely effective). Third, a series of words expressing a range of emotions was presented in random order and viewers were asked to tick/circle all those reflecting their response to the piece; the words were: ‘interesting’, ‘boring’, ‘stimulating’, ‘challenging’, ‘upsetting’, ‘disappointing’, ‘frightening’, ‘unexpected’, ‘anxiety-provoking’, ‘surprising’, ‘inspiring’, ‘beautiful’, ‘scary’, ‘fascinating’, ‘indifferent’, ‘moving’ and ‘thought-provoking’. Finally, viewers were invited to provide any further open-ended feedback about the installation in a free-text box, allowing the audience to unpack emotions that had emerged from experiencing the installation.

### Analysis

Descriptive statistics were used to characterise the study population. Categorical and ordinal variables are presented as counts or proportions. Words, phrases and sentences relating to expectations prior to viewing the piece and feelings after having experienced it were subjected to content analysis, where themes were first identified by two authors independently and a final list of themes agreed by discussion and consensus, bringing in a third author in the event that agreement could not be reached. Each response was then attributed to one of the themes so that the content analysis could be completed. The frequency of selection of each of the predetermined words relating to the viewers’ responses was also calculated. Differences in evaluation of the effectiveness of the piece in communicating the experience of heart transplantation and the complexity of cardiovascular anatomy were assessed using a Wilcoxon signed-rank test for paired data. The free-text comments were also thematically analysed, following a similar approach to that adopted with the words and phrases, where two authors independently coded the data and a final list of themes was agreed.

## Results

One-hundred and twenty-five people experienced the installation and all were invited to and completed the questionnaire. Study participants were predominantly female (86%) and based in the UK (86%). Viewers had a range of professional backgrounds, including academics (n=18), clinicians (n=16), artists (n=24) and other arts professionals (n=40). Three out of four viewers provided written feedback at the end of the survey. In a very small number of cases (n=5; 4%) viewers left before completing the second part of the questionnaire, thus we were only able to capture their expectations but not their response to the piece.

Viewers approached the piece with mixed expectations. Multiple themes were identified from the words/phrases/sentences. Table 1 presents the themes and the frequency of occurrence of each theme. Table 2 presents example words/phrases from dominant themes. Not all viewers provided three words, phrases or sentences and six participants explicitly said that they approached the piece with no expectations. In total, there were 326 words, phrases or sentences generated, while 346 words, phrases or sentences were provided by participants after having experienced the installation. Of the five viewers who, as mentioned, did not complete the second part of the survey, one specified that this was because their son had a ‘hole in the heart’ and the piece was ‘too close to home’. Themes from the second part of the survey are also summarised in table 1 with examples in table 2.

**Table 1:** Themes relating to the audience’s expectations (prior to viewing the artwork) and perceptions (after viewing the artwork). ‘Other’ refers to themes with counts of less than 5 (and typically only one)

Expectations before viewing		Perceptions after viewing	
Theme	Count (n)	Theme	Count (n)
Increased understanding	58	Emotion, connectedness, empathy	103
Patient experience, empathy, connection	47	Excitement, surprise, awe	56
Surprise and discovery	44	Insight and interest	42
Insight into cardiovascular disease, medicine	36	Beauty, preciousness, poetry	23
Inspiration and enjoyment	32	Medical, educational	20
Insight into arts-and-science process	31	Anxiety, discomfort, death	18
Visual, audio, visual and audio	26	Powerful, deep	17
To be moved	15	Intricate, complex, changeable	17
Relaxation, peacefulness	10	Music, sound, words	13
Beauty	7	Mindful, considerate	8
Anxiety, discomfort, sombreness	7	Calm, calming, warmth	6
Other	13	Other	23
Total	326	Total	346

**Table 2:** Example quotes for the five most commonly reported themes related to the audience’s expectations (prior to viewing the artwork) and perceptions (after viewing the artwork)

Theme	Example
Expectations before viewing	
Increased understanding	*‘to learn something new’, ‘increased understanding and awareness’, ‘learning about the heart*’
Patient experience, empathy, connection	*‘to get an insight into patient’s mindset’, ‘human story’, ‘voices of patients’, ‘insight into patients' response’*
Surprise and discovery	*‘joy of discovery’, ‘the unexpected’, ‘to be surprised’*
Insight into cardiovascular disease, medicine	*‘clogged arteries’, ‘heartbeat’, ‘fibrillation’, ‘the clinical gaze’*
Inspiration and enjoyment	*‘inspired’, ‘to be thought provoked’, ‘stimulation’, ‘shift my views’*
Perceptions after viewing	
Emotion, connectedness, empathy	*‘direct connection to [the] mother’s experience’, ‘human’, ‘feel it at an emotional level—especially the voice of the mother’*
Excitement, surprise, awe	*‘amazing’, ‘awe-inspiring’, ‘exciting’*
Insight and interest	*‘a real insight into [the] heart’, ‘stimulating’, ‘informative’, ‘in a new landscape—curious’*
Beauty, preciousness, poetry	*‘precious’, ‘beauty’, ‘poetic’, ‘poetry’*
Medical, educational	*‘healthcare surrounding’, ‘big space in the chest’, ‘makes you think how reliant we are on our bodies’, ‘terminology’*

**Figure 4:**
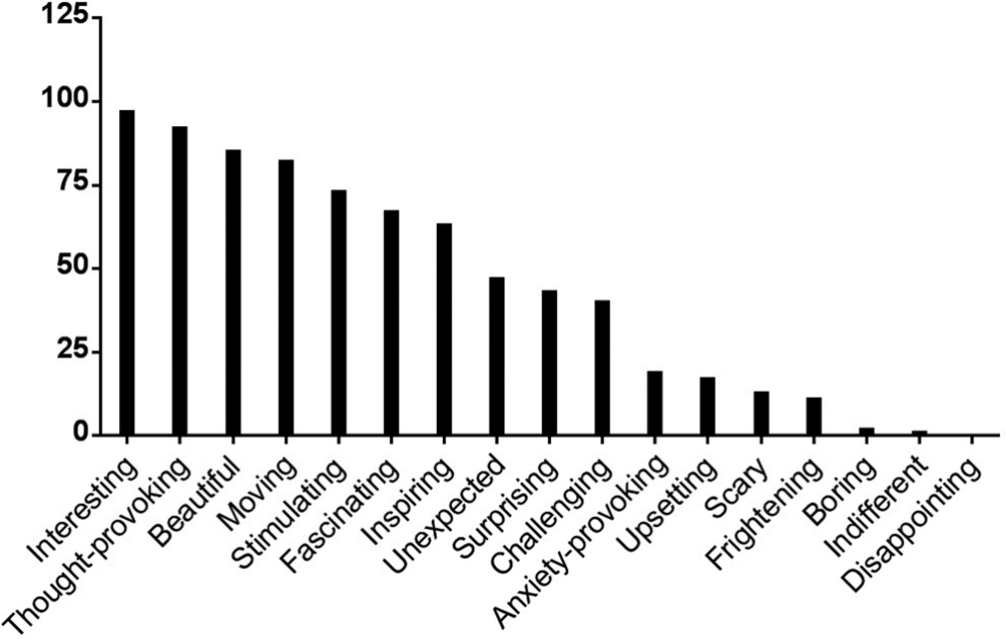
Summary of counts of predetermined words to describe participants’ responses to the installation.

Figure 4 provides information about the frequency of selection of the predetermined words. The median number of words circled was 6 (range 1–14).

With regard to the effectiveness of the piece, viewers found that the installation was more effective in communicating the experience of heart transplantation than in depicting the complexity of cardiovascular anatomy (heart transplantation: 8.0 (IQR: 7.0, 9.0); complexity of cardiac anatomy 6.5 (IQR: 5.0, 8.0); p<0.001, z=7.56).

Finally, a large amount of feedback was received, which in itself suggested that the viewers connected with and responded to the piece. Participants clearly enjoyed the experiential nature of the installation and while the comments tended to be brief, there were a number of themes that were evident. The emotional impact of the models and the soundtrack were highlighted by many of the viewers, with several using the word ‘moving’ to describe the impact. ‘*Overall very moving, evocative different experience*’; *‘Made me feel quite emotional as it made me realise how often I take my health for granted.’* For others, it encouraged a sharing of some of their own personal narrative, ‘*The story told by the mother brought together the everyday and the sublime—a bit like the discussion by the surgical staff attending the Caesarean birth of my first child*’, ‘*It reminded me of my own experience of my son’s open heart surgery—that was a hard time*’, ‘*It brought back memories, thoughts, raised questions about people I have known.*’

Viewers felt empathy towards the narrative and commented on the immersive nature of the piece: ‘*I would like to have known how the little boy got on*’, ‘*Wanted to experience more of the fear/worry/heart-ache from patients and families*’, ‘*I felt that I immersed in the setting and as If I was present when the mother was talking*’, ‘*You are on the edge of your seat. You can see the beating heart in your mind. You are afraid to ask where they are now but desperate to know.*’ Some also highlighted the intimacy of the installation and the privilege of sharing a story, particularly in relation to the soundtrack, *‘I felt special to be allowed to hear the very personal story of X’s mum and so be let ‘in’ to her intimate thoughts,’* with this intimacy enhanced by listening on headphones. The audience’s connection to the narrative in the piece was in fact strengthened by the sound element, with a number of participants describing its power: ‘*The sound part […] is the most powerful [and] gets you in much more than anything else*’; *‘I found the narrative about the little boy very powerful.*’ While some of the audience focused on the more medical aspects, for example, ‘*As a doctor, hearing [the] mum’s story gave me new insights into how much a marvel heart transplantation is*’, others were drawn more to the artistic elements, for example, ‘*I found the soundscape beautiful, enjoying the layering and interweaving of voice and heartbeat.*’ A number of participants also described the juxtaposition of different elements of the piece—*‘a patient’s story juxtaposed with the medical language*’, the juxtapositions of art and science, health and ill health, speech and music, music and vision, the personal and impersonal, recognising the effectiveness of such an approach in conveying the patient’s narrative.

The 3D models generated a lot of feedback, with some viewers fascinated by them—‘*Sculptures/models are very interesting [and] provoke interesting associations for me*’, ‘*I’ve never seen a model of a real heart and was surprised how different they look in shape and size*’, ‘*Reminiscent of the Victorian strange specimen, under glass clotches, untouchable, maybe repulsive, the hearts almost move within their spaces, organic*’ while others found them confusing or mentioned the need for more explanation—‘*I would like to see what kind of heart conditions are presented by each model [such that they would be] more educational and more visible for non-experts*’, ‘*More information on the different hearts*’, ‘*I would have appreciated some indication for help in how to ’read' the hearts—how to ’see' the ’defect' or the ’repair' more clearly in some way to add to the depth of my experience.*’ A number commented on the appearance of the models, seeking similarities with other more familiar objects, for example, they ‘*looked like bleached coral*’, ‘*become cathedral-like*’.

Although there were some conflicting views about whether there should have been more medical detail or less, whether there should have been music or whether that detracted from the installation, the prevailing sense was one of positivity about the piece and the thought-provoking and powerful nature of the overall experience.

## Discussion

This study looked at an audience’s response to a multimedia art installation that focused on congenital heart disease and heart transplantation. Different dimensions of heart disease such as its anatomy, treatment and technical language, as well as the unique stories of patients undergoing cardiac surgery and heart transplantation were explored in the piece. Such exploration was achieved by making use of different elements. At the level of cardiovascular anatomy, 3D printed models of heart disease are a medically accurate representation of patients’ hearts and their dimensions, intricacies and their respective differences in the presence of congenital defects and/or surgical corrections. At a narrative level, the inclusion of sound was central to the piece, mainly with the voices of a patient and his mother resulting in a strong storytelling component. At the level of medical language, the voice of the artist (again in the soundtrack) read technical names relating to conditions, medications and devices that pertain to the world being represented, and these were subtly echoed on the voiles of the hospital screens surrounding the table (figure 1). By creating such a layered landscape and having previously worked with patients and families in a participative manner,^6 18^ the artist essentially mediated the reality of those patients and those families, representing it as something new, different, beautiful, yet faithful to the underlying narrative and accurate with regard to the medical condition.

Viewers of *Making the Invisible Visible* approached the piece with mixed expectations, whether a desire to learn something novel about heart disease and heart transplantation, a curiosity for the dialogue between arts and science subtended in the piece, an interest for the patient’s experience, expecting the latter to emerge (in some unspecified way). From the arts-and-science collaboration perspective, it is interesting to explore viewers’ expectations as it could inform us about how the piece is framed (eg, with respect to interpretation materials), while remaining true to the intention of the artist. In this case, for instance, some viewers were fascinated by the heart models on display and captivated by their size. They drew interesting comparisons, but their feedback indicated that they would have liked additional information on basic anatomical features. This could have taken the form of a brief information sheet given to viewers once they had experienced the soundtrack, thus not influencing their experience but complementing it with simple additional medical notions. It is also interesting to note that the piece led to some extent to reification of the complex medical terminology incorporated in the soundscape and characterising treatment of heart disease, by giving it a form through the heart models.

Viewers responded to the piece on several levels. Of particular salience was how connected to the piece many viewers felt. Viewers commented on the mother’s account of her son’s heart transplantation, expressed their curiosity for how the story developed and even shared elements of their own narrative in the feedback. The direct inclusion of the patient and parent’s voice added to the honesty of the piece and to the ensuing sense of empathy. The artist met both patient and parent on the hospital ward and worked with them for a number of months, establishing a bond of trust. The inclusion of the patient’s voice was clearly a crucial element in the installation. Patients are often considered voiceless in a highly medicalised system.^24^ Inclusion of a soundtrack in the artwork gave real voice to the mother, to the young boy, to ‘the patient’. This element resonated greatly with the audience, and in a few isolated instances viewers found the piece ‘too close to home’, because of their own personal experiences of heart disease. While there can be anxiety about the potential to evoke such responses, this can in and of itself also provide valuable insights, although it is important to manage this ‘safely’. As part of our interdisciplinary practice we pay particular attention to the potential to elicit emotional responses in our audience, ensuring that audience members can be appropriately sign-posted to further support if they want this. The decision of the artist to give a voice to the mother of the patient is important, particularly—as remarked by Shapiro—in the light of a potentially unintended effect of patient-centred trends in medicine to somewhat delegitimise the patient’s voice, thus creating a tension with the advocated ‘narrative humility’ required on the part of professionals.^25^ In other words, the artwork can become a vehicle for the expression of stories that patients *need* to tell, and for them to tell such stories with their *own* language. Indeed, audience members also responded to the *tone* of the voice of the mother who spoke in the soundtrack, feeling that they were sharing an intimate moment with her but also being surprised that her tone sounded more joyful than they were expecting.

The role of the artist in the context of re-presenting illness experience is duple, on one level mediating accounts that have emerged through their participatory practice, and on another level making deliberate choices in the re-presentation, thus contributing to constructing meaning. With regard to the creation of an installation, it has been discussed by Farkhatdinov how the perception of these being made to be *experienced* rather than sold (in contrast with more commodified traditional art forms) contributes to the sense of community that they can engender in the audience, partly reflected in this case in the strong sense of empathy expressed by viewers.^26^

From a clinical perspective, artist-mediated narratives of illness could also represent a precious reflective stimulus. For student health professionals, for example, an approach including art making has been described for facilitating interpretation of cardiovascular anatomy and pathophysiology in natural and urban contexts, that is, moving beyond purely medical notions.^27^ More broadly, a formation which incorporates the narrative dimension has been discussed as a means of enabling students to critically appraise the implications of current approaches to training.^28^ Even beyond training, it has also been advocated that the arts can contribute to so-called ‘whole person understanding’ by providing insights into collective and individual experiences, as well as by augmenting the language of the practitioner.^28^ Although a subgroup analysis was not performed in the current study due to the relatively small sample size of the subgroups, the clinicians’ responses to the piece were illuminating in this regard and confirmed a positive impact. Clinicians remarked on the importance of the educational aspect but also identified how this kind of approach could be a good mechanism for communicating the personal experience of a disease. With regard to their own perspective, clinicians noted that the immersive nature of the installation helped to remind them about the patients’ understanding of a heart and their experience of cardiac problems and provided them with new insights into the wonders of heart transplantation. They also commented on the use of medical language, identifying that they, as clinicians, use medical terminology but that there is a person behind each of those clinical terms. Although these are isolated examples from one subgroup in the whole study population, the clinician perspectives reiterate the importance of further exploring the value of different forms of narrative work with health professionals. These observations are in line with previous work by Lapum *et al* exploring how a research-derived art installation influenced practice in the cardiovascular field, commenting on the ‘restorative power’ provided by the artistic approach and its knowledge transfer capacity in relation to both aesthetic and experiential domains.^17^

Interdisciplinarity is also essential and intrinsic to the work being presented. The dialogue between the artist, the bioengineer and the psychologist ensured that the piece had a scientific rigour in key elements such as the language or the heart models, without limiting the artist but instead aiming to ensure the truthfulness of the piece. The work also relied on a strong participatory process, with the artist working closely with patients and families on the hospital ward. This was reflected in the voice of patient and parent being incorporated in the soundtrack rather than being performed by an actor. This influenced the truthfulness of the representation and was reflected in the feedback, which emphasised the impact of the soundtrack and the mother’s account on the viewers’ experience and their interpretation of the piece. The interdisciplinary collaboration underpinning the piece also contributes to the novelty of the installation itself, where a technology such as 3D printing is directly employed to accurately portray the complexity of the medical condition(s) being explored.

Finally, an artwork cannot be viewed outside of its context, semantically and physically, and this certainly applies also to work in the art-and-medicine sphere. Brieber *et al* discussed how art museums foster a prolonged and more focused aesthetic experience versus a laboratory/clinical setting, demonstrating art experience and viewing behaviours in relation to the context where these occur.^29^ While *Making the Invisible Visible* was not targeted at a specific audience in its conception, the current setting was very specific to an art-and-medicine community and it would be interesting to explore responses to the piece in future (different) configurations.

### Limitations

This study may be at risk of selection bias, particularly because a high proportion of viewers had secondary education or higher. By presenting the work as part of an arts-and-health conference, many of the audience were possibly more curious and receptive towards this kind of representation than an audience from a different setting would have been. Conversely, it could be argued that a more specialised audience are also likely to have been more critical in their feedback. We did not have the opportunity to further explore individual reactions to the exhibition through more in-depth interviewing—for example, those of the audience member who felt the need to leave without completing the second part of the survey because of their personal connection with the content of the exhibition and the emotions it evoked. The audience was self-selective and the relatively small sample size precluded other interesting analyses such as exploring nuanced gendered responses or differences in perception in relation to the ethnic make-up of the audience. Future work, including future configurations of the piece, will pay particular attention to the mentioned biases and will aim to engage wider, more varied audiences.

## Conclusion

Audience response to a multimedia art installation representing the intense emotional journey of heart transplantation and the intricacies of cardiovascular anatomy in the presence of congenital heart lesions highlighted how this kind of representation can lead to an empathic response and an appreciation of the value and impact of illness narratives.
